# Exploring the Link between Premature Ovarian Insufficiency, Insomnia and Circadian Pathways

**DOI:** 10.5935/1518-0557.20240052

**Published:** 2024

**Authors:** Anna K. Kloster, Luana N. G. Adami, Mariana Moysés-Oliveira, Helena Hachul, Monica L. Andersen, Sergio Tufik

**Affiliations:** 1 Sleep Institute, Associação Fundo de Incentivo à Pesquisa, São Paulo, Brazil; 2 Departamento de Psicobiologia, Universidade Federal de São Paulo, São Paulo, Brazil

**Keywords:** sleep, insomnia, ovary, cortisol, hypothalamus, steroidogenesis, reproductive hormone

## Abstract

**Objective:**

To establish an interaction network for genes related to premature ovarian insufficiency (POI) and insomnia, and to identify biological processes that connect POI to the physiological clock.

**Methods:**

Previously reported lists of genes associated to POI and insomnia were contrasted and their intersection was used as input on protein-protein interaction analyses. POI-associated genes were contrasted with gene expression markers for neural circadian control and enriched pathways among their shared content were dissected.

**Results:**

The functional network generated from the intersection between POI and insomnia gene lists pointed to the central nervous system as the most relevant cellular context for this connection. After identifying POI-associated genes that play a role in neural circadian patterns, we observed the disruption of pathways related to the hypothalamic-pituitary-gonadal axis as the major genetic link between ovarian function and circadian neural circuits.

**Conclusions:**

These findings highlight neurological mechanisms that support the POI-insomnia interplay.

## INTRODUCTION

While the decline in oocyte quantity and quality is a normal physiologic occurrence as women age, some individuals experience diminished ovarian reserve much earlier than usual, and become prematurely infertile (Pastore *et al.*, 2018). Premature ovarian insufficiency (POI) is characterized by loss of regular ovarian function before 40 years of age and its etiology has been found to be associated with genetic and autoimmune factors (Ishizuka, 2021). Ovarian insufficiency is known to be associated with sleep disorders, such as insomnia (Ates *et al.*, 2022). Conversely, insomnia has a higher prevalence in females than in males, being present in approximately 33.3% of women (Lucena *et al.*, 2020; Tufik *et al.*, 2010).

The pathophysiology of insomnia is intrinsically linked to the hypothalamus, which orchestrates physiological regulatory mechanisms (Wen *et al.*, 2020), being crucial for the proper regulation of the circadian rhythm. The hypothalamus contains the suprachiasmatic nucleus (SCN), which has been considered as the ‘master pacemaker’ of the physiological clock in mammals, given the fact that it can generate self-sustained circadian rhythms and synchronize them daily in the entire body (Hastings *et al.*, 2018).

An interplay between the genetic architecture of insomnia and POI have been previously demonstrated, suggesting that there are common molecular mechanisms underlying this convergence (Moysés-Oliveira *et al.*, 2023). In a previous study, we have discussed the overlap between POI and insomnia-associated genes, which were implicated by large-scale populational genetics efforts (Moysés-Oliveira *et al.*, 2023). By dissecting the cellular processes that are connected to the genes commonly associated to both conditions, we suggested that cellular stress and cortisol release can induce oxidative stress, prompt DNA repair process, impair sleep quality and the adequate functioning of ovarian cells (Moysés-Oliveira *et al.*, 2023). Yet the specific circadian biological pathways which link those 2 clinical manifestations are still a knowledge void to the field, and the extent to which these circadian pathways affect neural circuits remains poorly understood. We performed novel *in silico* analysis to deepen our functional networks on the molecular patterns that connect POI and insomnia clinical manifestations.

## METHODS

### Manual curation of gene lists

Two sets of genes associated respectively with POI (total of 346 genes) and insomnia (total of 920 genes) were manually curated and a functional interaction network was established using as input the 27 genes that compose their intersection, as previously described (Supp. Table 1) (Moysés-Oliveira *et al.*, 2023). The list of genes associated with POI was contrasted to a third gene list, which was composed by markers for neural circadian control (total of 772 genes). The latter gene list was retrieved from recent single-cell RNA-seq studies which identified gene expression markers for the SCN and genes with circadian expression in neural cell types (Wen *et al.*, 2020).

### Protein-protein interaction network analysis

Protein-protein interaction (PPI) analysis was performed via String database (https://string-db.org/) with a minimal interaction score of 0.7, enabling a maximum of 5 additional interactors in the network. Protein clustering was defined using the Markov Clustering Algorithm (MCL) with an inflation value of 3.0. Besides, Cytoscape 3.9.1 was used for network visualization.

### Gene overlap analysis

Gene sets (i.e. genes associated with POI *vs*. markers for neural circadian control) were compared in order to obtain an intersection gene list. The GeneOverlap R package was used to conduct a Fisher’s Exact test, considering a total of 21,196 genes in the human genome with a statistical threshold of *p-value*<0.05.

### Pathway enrichment analysis

Enriched pathways were identified among gene lists of interest. In this analysis, Benjamini-Hochberg test, adjusting for multiple comparisons, was used to identify Gene Ontology (GO) and Kyoto Encyclopedia of Genes and Genomes (KEGG) enriched pathways, with a significance threshold of adjusted *p-value*<0.05.

## RESULTS

### Functional network for the POI and insomnia genetic intersection

**Figure 1:**
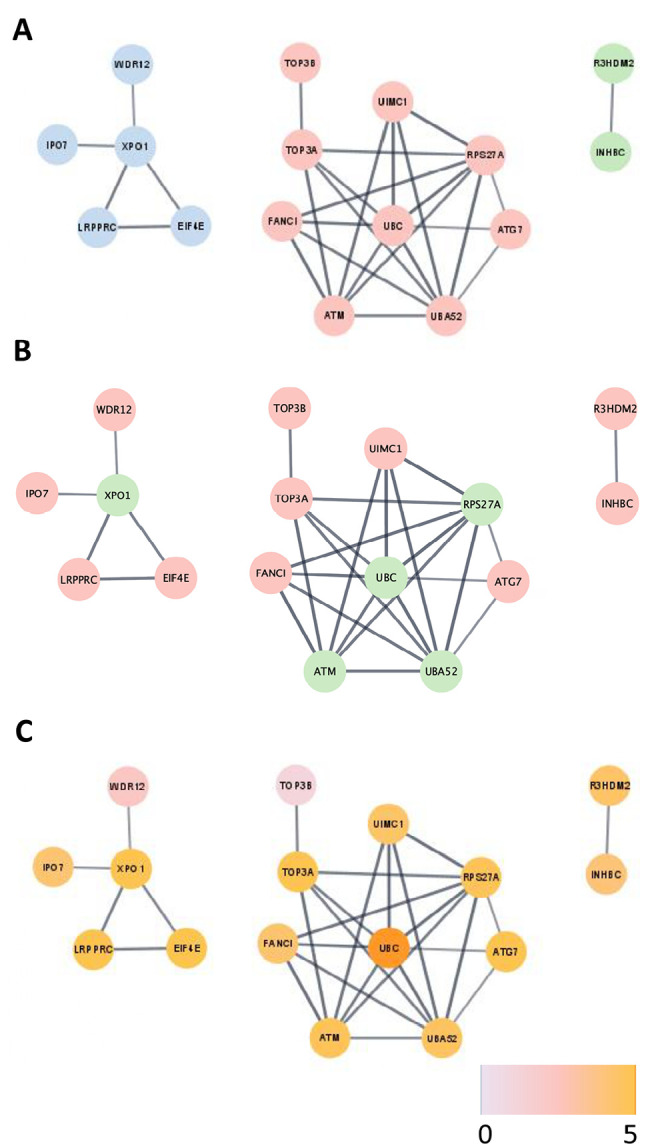
Functional network retrieved from insomnia and POI gene intersection A) PPI analysis retrieved 3 networks and a total of 16 nodes. Network 1 (blue) contains 5 nodes, including 4 proteins coded by genes in the intersect gene list. Network 2 (pink), contains 9 nodes, including 5 proteins coded by genes in the intersect gene list. Network 3 (green) contains 2 nodes, including 2 proteins coded by genes in the intersect gene list. B) The nodes in pink are part of the POI vs. Insomnia intersect gene list, the nodes in green are interactors. C) The color scale represents the expression level of these proteins in the nervous system in a scale from 1 to 5.

We have previously identified 27 overlapping genes between the gene lists associated with POI (346) and insomnia (920), indicating significantly more overlap than expected by chance (Figure 1, Supp. Table 1). The 27 intersection genes retrieved from the comparison between the insomnia and the POI gene lists based our first PPI analysis. This analysis formed 3 networks with a total of 16 nodes (Figure 1A), including 11 POI and insomnia-associated nodes plus 5 interacting nodes which were added to the network (Figure 1B). The great majority of those nodes (14 out of 16) are highly expressed in the central nervous system (Figure 1C).

After observing these results, we hypothesized that the SCN may be involved in the pathophysiology of the comorbidity between insomnia and POI, prompting us to investigate the relation between POI genes and the circadian control in the brain.

### Functional patterns for POI-associated genes related to neural circadian control

**Figure 2:**
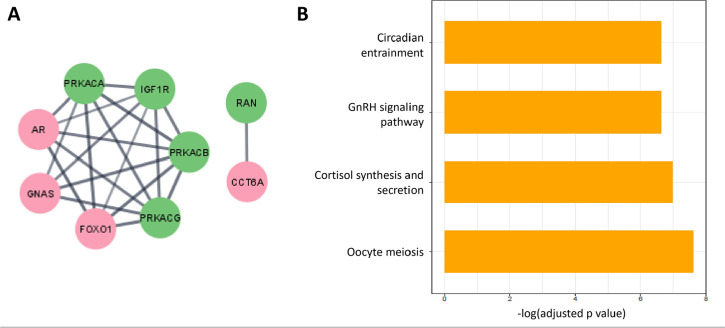
Functional network from the intersection between POI-associated genes and the list of genes related to circadian gene expression regulation in neural cell types. A) The nodes in pink are part of the POI vs. SCN intersection gene list, and the green nodes are the interactors. B) Representative enriched terms on the 9 intersect genes between POI-associated genes and gene list for neural circadian regulation. Pathways are ordered by their significance association [-log(adjusted p-value)].

We then contrasted the previously established list of POI-associated genes (346) against a list of markers for neural circadian control (772) (Supp. Table 1). An overlap of 9 genes was detected in this analysis (Figure 2, Supp. Table 1), and these interest genes were used as basis for second network analysis. In this analysis, a PPI network was built from the intersect between the POI-associated genes and the list of genes related to circadian gene expression regulation in neural cell types. The latter PPI analysis formed 2 networks with a total of 9 nodes (Figure 2A).

These 9 nodes were used as input to generate pathways enrichment analysis. The enriched pathways among POI-associated genes related to neural circadian regulation include: cortisol synthesis and secretion (adjusted *p-value*=1.26E-7, OR=261.38), circadian entrainment (adjusted *p-value*=2.51E-07, OR=171.17), ovarian steroidogenesis (adjusted *p-value*=9.91E-10, OR=541.98), GnRH signaling pathway (adjusted *p-value*=2.44E-07, OR=178.89), and oocyte meiosis (adjusted *p-value*=3.82E-8, OR=200.27) (Figure 2B).

## DISCUSSION

We observed that ovarian insufficiency and circadian traits share fundamental genetic and biological pathways involving neuronal circuity essential for sleep behavior, which is in agreement with the intrinsic relationship between the hypothalamic-pituitary-gonadal (HPG) axis and female reproductive functions. These neuronal circuits also control hormone release, which is essential for the physiology of the HPG axis (Carroll, 2007). After hypothalamic signaling, the pituitary gland promotes the induction of ovarian function, which releases hormones such as estrogen and progesterone. They are essential for follicular formation, induction, and oocyte maturation in the ovaries (Carroll, 2007), just as we can find in our enriched biological pathway, the oocyte meiosis pathway.

Synthesis and secretion of cortisol were found among the biological pathways observed as enriched in POI-associated genes related to neural circadian regulation. This observation can be explained by the fact that cortisol is one of the most relevant hormones involved in sleep biology and in insomnia pathogenic mechanisms, following a circadian pattern and being a marker of physiological stress. Cortisol changes are closely linked to alterations in gonadotropic hormones, which are essential for the female reproductive physiology function (Kim *et al.*, 2015). Concordantly, it has been demonstrated that women with POI have higher levels of fatigue and sleep latency (Benetti-Pinto *et al.*, 2019).

In animal models, circadian regulation of the HPG axis has received particular attention given the observation that radio frequency-induced lesions on SCN impair ovulation and produce a state of persistent estrus stage (Chappell, 2005; Raisman & Brown-Grant, 1977). Further investigations revealed that the SCN sends daily signals to the hypothalamus to prompt GnRH neurons to produce FSH and LH hormones through the pituitary. As observed, the GnRH signaling pathway is orchestrated by the SCN, which sends daily signals to the hypothalamus (Chappell, 2005). Under control of the hypothalamic GnRH and anterior pituitary FSH and LH, the ovaries manufacture estrogens and progesterones. The hypothalamic-pituitary-ovarian axis mediates the events of the ovulatory cycle, including follicular development, ovulation, corpus luteum formation, and menstruation. In addition, the estrogens and progesterone stimulate sexual desire and exert additional effects throughout in the body (Carroll, 2007), contributing to the enrichment of the ovarian steroidogenesis pathway found in this study. Researchers have recently found that clock genes are expressed at all levels in the HPG axis, which include the hypothalamus, the pituitary, and the ovaries (Sellix & Menaker, 2010). These clock genes seem to play an important role in the generation of synchronized pulsate hormone release (Chappell *et al.*, 2003), and in the gonad preparation for ovulation (Sellix & Menaker, 2010), endorsing the enrichment of the circadian entrainment pathway observed in our results.

The hypothalamic-pituitary-adrenal (HPA) axis influences many physiological functions, making an organism’s response to changes in the environment appropriate to its reproductive state (Oyola & Handa, 2017). Although the acute HPA response to stressors is a beneficial reaction, the constant activation of this circuit by chronic or traumatic stress episodes can lead to dysregulation of the HPA axis and cause pathology (Oyola & Handa, 2017). In comparison to males, female mice and rats exhibit a more robust HPA axis response, as a result of circulating levels of estradiol, which elevate stress hormone levels during non-threatening situations and during or after stressful factors (Oyola & Handa, 2017). Authors have associated insomnia with a global increase in ACTH and cortisol secretion, which are related to the regulation of the circadian pattern (Vgontzas *et al.*, 2001).

Our functional analysis indicate that the genes commonly affected by variants in the POI and insomnia architecture modulates the HPG response to stress stimuli, having implications in reproductive and neuronal processes. Our findings suggest that the disruption of pathways related to hormonal regulation is the major genetic link between ovarian function and circadian neural circuits. One of the major physiological mechanisms supporting the crosstalk between the ovary and the brain is the HPG axis. The intersect gene lists and the enriched pathways retrieved from our analysis might reflect how the circadian control of neural circuits affect the steroidogenesis and the female reproductive system.

Our results are in concordance with our hypothesis that cortisol release impacts sleep quality and ovarian function, and the genetic interplay between POI and insomnia involves the regulation of neural circadian pathways which are linked to stress response. These observations might represent a steppingstone towards personalized treatments tailored for women with POI and insomnia, based on their genetic variants of risk for these conditions.

## Appendix

**Supplementary Table 1:** POI, insomnia and neural circadian markers curated gene lists ^1^ (Di-Battista et al., 2020; Ruth et al., 2021) ^2^(Abel et al., 2020; Jansen et al., 2019; Rijo-Ferreira & Takahashi, 2019; Watanabe et al., 2022) ^3^ (Wen et al., 2020).

(1) List of POI related genes	(2) List of insomniarelated genes	POI and insomnia intersect genes	(3) List of neural circadian markers	POI and neural circadian markers intersect list
*AARS2*	*TRPC7*	*CDK2AP1*	GNAS	AR
*AIRE*	*DAB1*	*MON1A*	AVP	PRKACA
*ALOX12B*	*LEMD2*	*WDR12*	SYT1	IGF1R
*AMH*	*PTPRD*	*MLN*	MEF2C	PRKACB
*AR*	*PHF2*	*CARF*	DLK1	PRKACG
*ATG7*	*SNX29*	*LRPPRC*	PPP1R17	FOX01
*ATG9A*	*BTBD9*	*ATG7*	PEG10	GNAS
*ATM*	*NOL4L*	*R3HDM2*	CHODL	REN
*BMP15*	*CSMD3*	*POLR3H*	RORB	CCT6A
*BMPR1B*	*RNF123*	*INHBC*	CCDC136	
*BNC1*	*IP6K1*	*POLG*	FAM122B	
*BRCA2*	*OLFM4*	*IPO7*	HS6ST2	
*CDKN1B*	*TMEM161B*	*DENND1A*	FAM162A	
*CLPP*	*MEIS1*	*SBNO1*	TESC	
*CPEB1*	*CNTN5*	*BAG5*	VGF	
*CSB-PGBD3*	*FOXP2*	*FANCI*	RPS14	
*DACH2*	*SDK1*	*TOP3A*	DBI	
*DIAPH2*	*RBFOX1*	*SLC25A12*	SLIT2	
*DMC1*	*DCC*	*TOP3B*	RASD1	
*EIF4ENIF1*	*KDM4B*	*EIF4E*	PLCD4	
*ESR1*	*SEMA3F*	*UIMC1*	PRNP	
*FANCA*	*STRBP*	*RREB1*	APBB2	
*FANCL*	*PDE2A*	*HLA-B*	RASGRF1	
*FANCM*	*IP6K3*	*ELAVL2*	SH3KBP1	
*FIGLA*	*GLIS3*	*CNNM2*	PCLO	
*FMR1*	*FAM198A*	*LYG1*	LCORL	
*FOXL2*	*SNCA*	*PPM1F*	AVPI1	
*FSHR*	*UQCC2*		NMS	
*GDF9*	*C12orf65*	2010204K13RIK		
*HARS2*	*TCF12*		RASA4	
*HFM1*	*RBM5*		RAB3B	
*HSD17B4*	*FES*		CYGB	
*IGSF10*	*GPR139*		PDE2A	
*INHA*	*DCAKD*		THSD7A	
*KHDRBS1*	*RBM4*		TUBA1A	
*LARS2*	*GNAO1*		ZBTB20	
*LHCGR*	*FAM120A*		SCN9A	
*LHX8*	*TM9SF4*		MXD4	
*LRPPRC*	*FOXP1*		LIAS	
*MCM8*	*PURG*		CD63	
*MCM9*	*EFNA5*		RORA	
*MEIOB*	*CAMKV*		MTPN	
*MND1*	*RILPL2*		MID1IP1	
*MRPS22*	*BSN*		PMM1	
*MSH4*	*ZBTB26*		RPL18	
*MSH5*	*ASCC3*		1700037H04RIK	
*NANOS3*	*MST1R*		LDHA	
*NOBOX*	*TRAIP*		ZFAND6	
*NOTCH2*	*SPI1*		DNAJC21	
*NR5A1*	*DIAPH3*		GDA	
*NUP107*	*ITPR3*		VIP	
*PGRMC1*	*ASXL1*		PPP1R1A	
*PMM2*	*LIN28B*		CHGA	
*POF1B*	*FAM172A*		C1QL3	
*POLG*	*RBM6*		SYT10	
*POLR2C*	*MON1B*		ATP1B1	
*POLR3H*	*NMT1*		IGFBP5	
*PSMC3IP*	*UNC5D*		CADPS2	
*PTHB1*	*BRINP3*		CALB2	
*SALL4*	*SNF8*		PAM	
*SF1*	*SALL1*		HBEGF	
*SGOL2*	*CHCHD3*		RASL11B	
*SMC1β*	*MTA3*		IQSEC3	
*SOHLH2*	*MLLT10*		LHX1OS	
*SPIDR*	*CDK2AP1*		SIX3OS1	
*STAG3*	*KIF3B*		POU2F2	
*SYCE1*	*RABGAP1*		LHX1	
*TGFBR3*	*CCDC148*		OCIAD2	
*TP63*	*RC3H2*		EPHB1	
*TRIM37*	*CDHR4*		STK32A	
*TWNK*	*CCDC36*		ADCY3	
*WDR62*	*MPHOSPH9*		CAMK2D	
*WT1*	*MON1A*		SLC2A13	
*WWTR1*	*PDE4B*		GPX3	
*ABHD12*	*SLC39A13*		VAT1L	
*ACACA*	*SYCE1L*		GAD1	
*ACSBG1*	*NTM*		WBP5	
*ACSL1*	*ACTN3*		PCDH19	
*ACSL3*	*KCNIP4*		CHST1	
*ADNP*	*PIK3C2A*		PBX1	
*ADSS*	*GRIA1*		VSNL1	
*AFF3*	*CDH8*		NRSN1	
*AL162431.1*	*MED27*		GRIK1	
*ANAPC4*	*UGP2*		RGS16	
*ANKRD30A*	*RBM14-RBM4*		GPD2	
*APOLD1*	*HS6ST3*		SLC12A2	
*APTX*	*UBE2Z*		PARM1	
*ARHGEF18*	*FURIN*		TPPP3	
*ARID3A*	*IGF2BP1*		CDK5R1	
*ARID3B*	*SERGEF*		SLC24A3	
*AS1*	*ASXL3*		HMGB3	
*ATCAY*	*CCDC71*		UBL3	
*ATXN8OS*	*ZBTB6*		PDCD4	
*BAG5*	*TOX3*		SPOCK2	
*BCL2*	*ATP2B2*		FLRT3	
*BCL2L11*	*CCS*		FUT9	
*BIRC6*	*BOLL*		NPAS4	
*BLK*	*PSMC3*		CEBPG	
*BLNK*	*FYN*		CCK	
*BMP4*	*CDH13*		SCG2	
*BOD1L1*	*RBM4B*		PROKR2	
*BRCA1*	*TCF4*		4930426D05RIK	
*BRD3*	*PLCL1*		NSMF	
*BRE*	*NUCB2*		NDRG1	
*BTBD1*	*ZNF580*		PRR5L	
*CALCR*	*HCK*		COL25A1	
*CARF*	*SAMD5*		CST3	
*CASC22*	*RAB1B*		PEA15A	
*CBFA2T3*	*KLF7*		VIPR2	
*CCNT2*	*WDR90*		MRPL41	
*CCT6A*	*VWC2*		LBH	
*CDIP1*	*TMX2*		ALCAM	
*CDK2AP1*	*COG5*		GSTA4	
*CHAMP1*	*ZNF865*		ZIC1	
*CHD7*	*PLCH1*		B3GAT2	
*CHEK2*	*FAM222B*		CDH13	
*CIT*	*SERPING1*		GAS7	
*CNNM2*	*DDX39B*		TLE4	
*CPEB4*	*STAU1*		GADD45G	
*CPNE1*	*CCDC178*		ADD3	
*CTD-2085J24.4*	*HBP1*		KHDRBS2	
*DCAF4L1*	*SLC35B4*		NTM	
*DDIT4L*	*NEGR1*		ATP2A2	
*DDX5*	*DOCK3*		GRIA1	
*DEFB125*	*C10orf11*		MSMO1	
*DENND1A*	*ROBO2*		CAMK1D	
*DEPTOR*	*RAPGEF2*		PRKCA	
*DIDO1*	*ARHGAP15*		ZIC4	
*DMRT1*	*BBS1*		DGKG	
*DNA2*	*RPL4*		SLC39A6	
*DNAJC11*	*LUZP2*		NOS1AP	
*DNAJC7*	*MOB4*		TSHZ2	
*DNER*	*ZWILCH*		SYT17	
*DPPA3*	*SLC4A10*		AKAP17B	
*EAPP*	*ZDHHC24*		GABRG3	
*EBF3*	*MAP2K1*		TACC2	
*EFHC2*	*SPTBN2*		FOSB	
*EIF4E*	*SETD8*		EDIL3	
*EIF4EBP1*	*ZPBP*		MYO10	
*ELAVL2*	*FGF14*		JAM3	
*ENTPD1*	*TMEM219*		SQLE	
*ERBB2*	*EXD3*		LRP8	
*ERLIN1*	*VWC2L*		DBPHT2	
*ESRP1*	*ACBD4*		KLF6	
*ESYT2*	*SNAPC5*		HMGCS1	
*ETAA1*	*WDR12*		GRP	
*ETS1*	*GNAT1*		SYNPR	
*EXO1*	*FIBCD1*		CD24A	
*FAAP24*	*ICA1L*		SPOCK3	
*FAM172BP*	*SYT14*		PCP4	
*FAM208B*	*NLRP9*		TPBG	
*FAM83F*	*MMRN1*		LIN7A	
*FANCB*	*KIF2A*		PPFIA2	
*FANCI*	*UBE3B*		CBLN2	
*FBXL17*	*FRS3*		PCDH9	
*FGFRL1*	*AGAP1*		NGB	
*FIGNL1*	*MLN*		PCSK1N	
*FLJ26245*	*RHOT2*		TENM2	
*FOXO1*	*GIP*		GABRG1	
*FSHB*	*GALNT2*		FXYD7	
*FTO*	*MSRA*		GAP43	
*GAB2*	*B3GAT1*		GABRA2	
*GBAS*	*TANC2*		PLCB1	
*GCKR*	*C5orf30*		ARHGAP6	
*GEMIN5*	*TRAF4*		RPRM	
*GJA1*	*KCTD10*		CTHRC1	
*GK*	*NPAS3*		SLC12A5	
*GMPR*	*IPO11*		TPD52L1	
*GNAS*	*RBM14*		MATK	
*GORASP2*	*KCTD13*		RBFOX3	
*GPCPD1*	*ATP5G1*		GABRB1	
*GSPT1*	*BAZ2B*		TMEM91	
*GSTM4*	*IQCK*		KLHL13	
*GTF2A1L*	*MCCD1*		TENM4	
*HCAR2*	*ZC3H7B*		PTPRD	
*HEATR3*	*SORCS3*		RAB3C	
*HELB*	*ARFGEF2*		PRKCE	
*HELQ*	*DPP3*		NSG2	
*HIST1H2BJ*	*NFE2L1*		SH3GL2	
*HLA-B*	*RHOD*		AR	
*HLA-DQA1*	*COPZ1*		TCAF1	
*HLA-E*	*ZBTB37*		TIMP2	
*HOXD4*	*CBX1*		KCND2	
*IGF1*	*MST1*		NRP1	
*IGFBP1*	*TAOK2*		CAMK2N1	
*IKZF2*	*FBXL16*		PPP3CA	
*ING4*	*MBNL1*		MAGI1	
*INHBB*	*PELI3*		JUNB	
*INHBC*	*DNM1*		D930028M14RIK	
*INO80*	*ZDHHC5*		BDNF	
*IPO7*	*SLC38A3*		IRS4	
*IPO8*	*MFSD1*		PTN	
*ITIH3*	*HEXIM2*		DDC	
*KCNQ1*	*CTNND1*		RPH3A	
*KDELR3*	*ZNF784*		PKIB	
*KLF10*	*LYRM1*		OLFM1	
*KNL1*	*RAPSN*		CELF4	
*KPNA3*	*MAP3K9*		AHI1	
*L3MBTL3*	*HTR1F*		NRXN3	
*LBR*	*PPA2*		MGAT4C	
*LINC00824*	*NRG3*		NAP1L5	
*LRRC32*	*ZNF521*		RMST	
*LYG1*	*DUPD1*		SLC24A2	
*MAFTRR*	*C3orf84*		SYN2	
*MAP2K2*	*DPH7*		KHDRBS3	
*MBTPS2*	*ZMYND19*		PPM1L	
*MCM10*	*CACNA2D2*		NEGR1	
*MICA*	*CSMD1*		ADCY2	
*MIR3977*	*RASGEF1C*		SIX6	
*MLN*	*NCAPG*		PKP4	
*MON1A*	*PDCD11*		PAK1	
*MRPS31*	*UHRF1BP1*		CXXC5	
*MSH6*	*GPRC5B*		BCL11A	
*MSI2*	*ZFHX4*		ZCCHC17	
*NADK2*	*RFTN2*		CALM3	
*NAMPT*	*STOML1*		1110004F10RIK	
*NDUFV3*	*MED12L*		DPYSL3	
*NLRP11*	*RGS17*		EPHA4	
*NLRP4*	*CARF*		SEPW1	
*NMRK2*	*SNX11*		4930447C04RIK	
*NSUN4*	*KCNMB2*		TUBB2A	
*NUP62*	*PITPNM2*		MRAP2	
*OOEP*	*STOX1*		PNMA3	
*OPA1*	*HAAO*		ABCA5	
*OR5V1*	*HSPE1-MOB4*		ABCD3	
*PAPD7*	*ASPHD1*		ABI3BP	
*PARL*	*TET1*		ACP6	
*PELO*	*C15orf59*		ACSS1	
*PEX5*	*LRPPRC*		ACTN1	
*PFKM*	*MVK*		ADAMTS20	
*PIWIL1*	*VEGFA*		ADGB	
*PMF1*	*MYO1H*		ADGRB1	
*PNKP*	*RET*		ADGRB2	
*PNP*	*PLAGL2*		ADGRG1	
*PPARG*	*USP49*		ADGRL3	
*PPIH*	*TCF7L2*		ADK	
*PPM1B*	*MMAB*		ADORA1	
*PPM1F*	*DCAF16*		AGRN	
*PPP1CB*	*ARRDC1*		AIFM3	
*PPP2R3A*	*C11orf31*		AKAP8	
*PPP5C*	*PLEKHA7*		ALDH7A1	
*PQLC1*	*KMT2A*		ALKBH2	
*PRIM1*	*MAPKAPK3*		AMBRA1	
*PRKDC*	*TET2*		AMHR2	
*PRR3*	*BAK1*		ANGPTL4	
*PRRC2A*	*FAM57B*		ANKS1B	
*PTPRJ*	*KLHDC8B*		ANP32A	
*PVT1*	*ATG7*		AP1M1	
*R3HDM2*	*PLCD3*		APIP	
*RAD18*	*P2RY12*		APLP1	
*RAD51*	*PCLO*		ARF5	
*RAD54L*	*PRMT6*		ARL1	
*RASAL2*	*ABCC8*		ARL6IP1	
*RBBP8*	*RPS13*		ARMCX2	
*RBM25*	*NICN1*		ASB8	
*RBM26*	*C6orf106*		ATOH8	
*RBM46*	*CCDC77*		ATP2C1	
*REV3L*	*RNF130*		ATP5G2	
*RHBDL2*	*GRM5*		ATP5SL	
*RIBC2*	*CTDSP2*		AVPR1A	
*RIF1*	*ASTN2*		AZIN1	
*RNF220*	*PRKAR2B*		B230303O12RIK	
*RNF44*	*BAG6*		BACH1	
*RPA1*	*DUSP13*		BAZ2A	
*RPAIN*	*C16orf62*		BC030336	
*RREB1*	*PRICKLE4*		BGN	
*RTEL1*	*MYBPC3*		BLCAP	
*RUNX1*	*APOE*		BLMH	
*SBNO1*	*TIPIN*		BLOC1S5	
*SCAP*	*NUP160*		BMP1	
*SCCPDH*	*CTNNA3*		BOLA2	
*SERAC1*	*ADGRB3*		BORCS5	
*SH3PXD2B*	*CTSF*		BRMS1	
*SIRT1*	*P2RY14*		BTBD17	
*SLC25A12*	*MICB*		C1GALT1C1	
*SLC9A8*	*SNRPC*		CABLES1	
*SLCO4A1*	*AGBL2*		CACNA1H	
*SLMAP*	*DEFB124*		CADM1	
*SNTA1*	*RANGAP1*		CALR	
*SOD2*	*CYB561*		CAPN2	
*SPATA2*	*SMAD5*		CASKIN1	
*SPRTN*	*DNAH11*		CCDC18	
*SPRY4*	*LOC81691*		CCDC25	
*SPTSSA*	*PAM*		CCDC50-PS	
*SRP9*	*LAMB2*		CCDC66	
*SRPK1*	*CCAR1*		CCNYL1	
*STON1*	*UBA7*		CCT2	
*SV2A*	*DUSP6*		CCT6A	
*SYCP2L*	*MEI1*		CD276	
*TBC1D1*	*PEBP4*		CDC25A	
*TDRD3*	*R3HDM2*		CDC42SE1	
*TFAP2C*	*CCDC134*		CDK9	
*TGFBR2*	*PCDH1*		CDYL	
*TIPARP*	*PHLPP1*		CELSR2	
*TLK1*	*DOC2A*		CENPU	
*TMEM120B*	*COPZ2*		CEP83	
*TMEM169*	*HOMER2*		CHCHD1	
*TMEM86B*	*SEC11C*		CHKB	
*TNPO3*	*PCGF6*		CIART	
*TNRC18*	*DUSP15*		CIRBP	
*TOP3A*	*TSTA3*		CLDN10	
*TOP3B*	*FNBP4*		CMTM3	
*TP53BP1*	*DIS3L*		CMTR2	
*TRA2A*	*RHOA*		CNPY3	
*TRIM33*	*PHF7*		CNTRL	
*TSC22D2*	*TPH1*		COLGALT1	
*U2SURP*	*ARHGEF7*		COPS7A	
*UBE2L3*	*POLR3H*		CORO1C	
*UBXN2A*	*PKP4*		COX4I2	
*UFD1L*	*HIVEP3*		CP	
*UIMC1*	*FAM173A*		CPEB1	
*UPRT*	*TTLL6*		CPEB2	
*UQCRC1*	*ERI3*		CPLX2	
*USP3*	*N6AMT2*		CPSF3	
*USP34*	*GABBR1*		CPT1C	
*USP7*	*NFE2*		CREB1	
*USPL1*	*ALDOA*		CRELD2	
*WASIR2*	*ARSG*		CSF2RA	
*WDR12*	*IQCH*		CSMD1	
*WNK1*	*COPS7A*		CSPG5	
*WRB*	*ZBTB20*		CTPS	
*WWOX*	*PHF14*		CTSA	
*YBX2*	*TOB2*		CUEDC1	
*YBX3*	*SGSM2*		CUL9	
*YDJC*	*PLEKHM2*		CUX1	
*YY2*	*ATP1B1*		CX3CL1	
*ZAR1*	*ZNF664*		D16ERTD472E	
*ZCCHC2*	*TRDN*		D730045B01RIK	
*ZCCHC8*	*LINGO2*		DAZAP2	
*ZFP42*	*KCNG3*		DBP	
*ZFPM1*	*INHBC*		DCLK2	
*ZNF208*	*DNAH1*		DCLRE1C	
*ZNF275*	*DCUN1D3*		DDHD2	
*ZNF518A*	*C2orf47*		DDX3Y	
*ZNF638*	*TEX10*		DHCR7	
*ZNF704*	*INVS*		DHDH	
*ZNF728*	*BTBD7*		DIAPH3	
*ZNF91*	*CD101*		DNAH7C	
*ZRANB1*	*C3orf62*		DNAJC3	
*ZXDC*	*GPR21*		DPM2	
*MBTPS2 / YY2*	*TMEM64*		DUSP16	
*SLL4*	*UNC79*		DZIP1L	
*ERCC6*	*P2RX3*		E130215H24RIK	
*SOHLH1*	*RBM39*		E130311K13RIK	
*AMHR2*	*NRG1*		EFHD1	
*IL11*	*RASGRF2*		EIF5	
	*KSR2*		EMG1	
	*ERI2*		EP400	
	*RASGEF1A*		EPB41L5	
	*OR2J2*		EPN1	
	*KLC2*		ERCC1	
	*FNIP2*		ERCC2	
	*ERAL1*		ETHE1	
	*GREB1L*		F3	
	*KIF5A*		FABP7	
	*CSDC2*		FAM107A	
	*SOX5*		FAM120B	
	*CAMKMT*		FAM136A	
	*SEMA3G*		FAM160B2	
	*XPO4*		FAM20B	
	*PRKN*		FAM214A	
	*C15orf40*		FAM222A	
	*CASC10*		FAM45A	
	*MYO16*		FAM84A	
	*GIN1*		FAM96A	
	*UBR5*		FBXL12OS	
	*MACROD2*		FBXL3	
	*ATAD5*		FBXO17	
	*TCTA*		FERMT2	
	*MMS22L*		FGFR2	
	*WDR82*		FGFR3	
	*PACS1*		FHOD3	
	*LCTL*		FJX1	
	*EIF4E3*		FKBP8	
	*PCDH12*		FOXJ2	
	*AKT3*		FOXO1	
	*ACTN2*		FOXO3	
	*SLC7A5*		FZD5	
	*GPR22*		FZD7	
	*YPEL1*		FZR1	
	*KDM5A*		G6PC3	
	*ASCL1*		GALT	
	*SERPINC1*		GCDH	
	*XPNPEP1*		GDPD2	
	*BEND7*		GGT7	
	*PCDH9*		GLTSCR1	
	*PICALM*		GLUL	
	*NHP2L1*		GM10419	
	*EYS*		GM10714	
	*RORB*		GM10827	
	*PHF5A*		GM11868	
	*PDCL2*		GM12758	
	*C5orf28*		GM12791	
	*SRRM2*		GM13436	
	*TWF2*		GM13736	
	*QRICH1*		GM14450	
	*HEXIM1*		GM14630	
	*TAL1*		GM15432	
	*RASGRP1*		GM15500	
	*CCDC78*		GM20515	
	*NCR3LG1*		GM24379	
	*C1QTNF4*		GM26910	
	*UGGT2*		GM28151	
	*KLC1*		GM29199	
	*EFCAB13*		GM36963	
	*HMGA2*		GM37174	
	*PACRG*		GM37274	
	*HOXB3*		GM42829	
	*CNTNAP5*		GM43254	
	*CPNE7*		GM43343	
	*POLG*		GM43462	
	*ANKFY1*		GM43681	
	*ARAP1*		GM44421	
	*CLOCK*		GM44432	
	*UHRF1*		GM4953	
	*U2AF2*		GM5141	
	*MDGA1*		GM5607	
	*MYO18A*		GM5652	
	*INO80E*		GM5841	
	*C16orf72*		GM6467	
	*PTK2*		GM7132	
	*LGALS3*		GM715	
	*RANBP17*		GM8054	
	*HNRNPUL2*		GM8394	
	*DMXL2*		GM8643	
	*DAG1*		GM9333	
	*TEFM*		GM996	
	*DEFB115*		GPC1	
	*NADK*		GPT2	
	*DNAJC1*		GRIK4	
	*GNL1*		GRINA	
	*SLC39A8*		GSR	
	*ADARB1*		GUCY1A3	
	*LHFP*		HACD4	
	*HLA-G*		HAUS3	
	*TTC36*		HERC4	
	*IPO7*		HERPUD1	
	*SLC22A23*		HIPK1	
	*PAIP1*		HIST1H4J	
	*HSPA12A*		HNRNPC	
	*SP4*		HNRNPL	
	*KNOP1*		HSP90AB1	
	*NOS1*		HSP90B1	
	*USP20*		HSPA2	
	*PPP4C*		HSPA4L	
	*ZNF420*		HSPA5	
	*MED19*		HSPA8	
	*C1QTNF7*		HSPH1	
	*RIMS3*		HTATSF1	
	*PSMD14*		ID1	
	*SORBS2*		ID3	
	*PAX6*		ID4	
	*DIMT1*		IDS	
	*RBL2*		IFT74	
	*ASB1*		IGF2R	
	*THUMPD1*		IMMT	
	*INA*		INPP5B	
	*ZMYND8*		ITIH3	
	*EP300*		ITPKC	
	*NCKIPSD*		JAM2	
	*AVL9*		JMY	
	*DENND1A*		JTB	
	*SPAG5*		KARS	
	*CCDC92*		KCNA2	
	*TMEM106B*		KCND3	
	*TBL2*		KCNJ3	
	*RPP21*		KDSR	
	*OGFOD2*		KIF5B	
	*POPDC3*		KIRREL3	
	*ADCY9*		KLC4	
	*CYB5D2*		KLF7	
	*TRIM27*		KLF9	
	*NPAS4*		KLHL21	
	*CD3D*		KMT2B	
	*SKAP1*		KY	
	*GABRB2*		LAP3	
	*PAX5*		LASP1	
	*MLF2*		LENG8	
	*METTL16*		LEPROTL1	
	*NFASC*		LFNG	
	*SH3TC2*		LGR6	
	*TEF*		LHFPL2	
	*STAB1*		LIMCH1	
	*RTN4*		LIN7C	
	*NCAM1*		LRRC58	
	*SBNO1*		LY6C1	
	*FBXL13*		LY6G6D	
	*ADCK1*		LY6H	
	*WDR24*		LYSMD2	
	*SLC23A3*		MAML1	
	*AS3MT*		MAPK1	
	*TRAF3IP1*		MAPT	
	*NPY*		MAT2A	
	*TSPAN5*		MAX	
	*OXR1*		MCL1	
	*LYRM2*		MCMBP	
	*LDB2*		MCPH1	
	*GYLTL1B*		MEAF6	
	*PRRG2*		METRN	
	*SMARCC1*		MFAP2	
	*SKIL*		MFSD2A	
	*TTLL11*		MICAL1	
	*SNW1*		MIEF2	
	*TYW5*		MIR9-3HG	
	*ACSM3*		MIRG	
	*ARIH2*		MLEC	
	*CHADL*		MMP14	
	*JMY*		MOB3C	
	*BSCL2*		MOB4	
	*LCORL*		MPC2	
	*RORA*		MPP1	
	*CALB2*		MPZL1	
	*NBEAL1*		MRPL21	
	*GLO1*		MRPL23	
	*USMG5*		MRPS22	
	*EYA2*		MRS2	
	*RNF19B*		MSI1	
	*FOXE3*		MTX3	
	*FGGY*		N4BP3	
	*INADL*		NAA40	
	*IL23R*		NBN	
	*CRYZ*		NDEL1	
	*FUBP1*		NDUFV3	
	*GTF2B*		NEDD1	
	*WDR3*		NFKBIA	
	*RPRD2*		NIN	
	*AQP10*		NISCH	
	*MAEL*		NKX6-2	
	*RGS16*		NOTUM	
	*ACBD3*		NPRL3	
	*SFMBT2*		NR1D1	
	*ARHGAP21*		NR1D2	
	*EGR2*		NRARP	
	*NRBF2*		NT5M	
	*VDAC2*		NTHL1	
	*BTRC*		NTSR2	
	*NT5C2*		NUDT16	
	*WDR96*		NUDT19	
	*CCKBR*		NUDT8	
	*ZNF143*		OLFR287	
	*DNAJC24*		ORMDL3	
	*NDUFS3*		OSBPL1A	
	*MTCH2*		P4HA1	
	*CLP1*		PAFAH1B1	
	*METTL12*		PAFAH2	
	*EED*		PANK1	
	*CTSC*		PAPSS1	
	*GXYLT1*		PAQR6	
	*TUBA1B*		PAQR8	
	*TUBA1A*		PARP3	
	*TUBA1C*		PCCB	
	*LEMD3*		PCNX3	
	*ATXN7L3B*		PDE4D	
	*BRAP*		PDE4DIP	
	*FBXO21*		PDIA3	
	*DNAH10OS*		PER1	
	*B3GALTL*		PER2	
	*CLDN10*		PFKFB4	
	*STK24*		PGAM5	
	*TEX29*		PGM2	
	*SSTR1*		PGS1	
	*VRK1*		PIAS3	
	*BAG5*		PIGA	
	*SPATA5L1*		PIGT	
	*SEMA6D*		PIK3C2B	
	*ONECUT1*		PIP4K2A	
	*MAP2K5*		PIP5K1C	
	*PML*		PLCE1	
	*KIAA1199*		PLD1	
	*FANCI*		PLEKHD1	
	*SLCO3A1*		POLA1	
	*IGF1R*		POLE4	
	*STUB1*		PPARD	
	*TCEB2*		PPCS	
	*SLX1B*		PPFIBP1	
	*MAPK3*		PPOX	
	*GOT2*		PPP1R15B	
	*ZNRF1*		PPP3R1	
	*LDHD*		PRDM9	
	*CNTNAP4*		PRDX6	
	*FBXO31*		PRPF40B	
	*TSR1*		PSMD3	
	*SHISA6*		PSMD7	
	*TOP3A*		PSPH	
	*DHRS13*		PTER	
	*NF1*		PTPRR	
	*HOXB4*		PUF60	
	*PSMC5*		PXDN	
	*BPTF*		PYGO2	
	*SDK2*		QSER1	
	*ARHGDIA*		QSOX1	
	*ASPSCR1*		RAB34	
	*RAC3*		RABEPK	
	*MEX3C*		RALGPS2	
	*KCNG2*		RANBP9	
	*PRDX2*		RAP1GAP	
	*RNASEH2A*		RARRES2	
	*SYCE2*		RASA2	
	*FARSA*		RAVER2	
	*CALR*		RB1CC1	
	*GATAD2A*		RBM27	
	*USF2*		RBM4	
	*PAK4*		RDH5	
	*A1BG*		RECQL	
	*TRIM28*		REEP4	
	*MYT1L*		REXO2	
	*OXER1*		RFXANK	
	*BCL11A*		RFXAP	
	*ACTR1B*		RHBDD1	
	*DDX18*		RIDA	
	*CCDC93*		RIF1	
	*MARCO*		RMDN2	
	*ORC4*		RMND5B	
	*TANK*		RNASEH2B	
	*B3GALT1*		RNF11	
	*DYNC1I2*		RNF115	
	*SLC25A12*		ROCK2	
	*ATP5G3*		RP23-316L10.2	
	*AGPS*		RP23-425N12.2	
	*C2orf69*		RP24-246F6.2	
	*PCSK2*		RP24-77E13.10	
	*XRN2*		RPE	
	*FRG1B*		RPL36A	
	*NOL4L-DT*		RPS6KC1	
	*CD40*		RSAD1	
	*ZBTB46*		RUNDC3A	
	*OPRL1*		RUVBL2	
	*TOP3B*		RXRG	
	*TOMM22*		SASH1	
	*CBX6*		SCN2B	
	*PMM1*		SDC4	
	*SEC13*		SDF2L1	
	*VGLL4*		SDF4	
	*THRB*		SDHAF2	
	*USP4*		SELT	
	*CGGBP1*		SEMA4A	
	*IFT57*		SENP5	
	*MYH15*		SEP15	
	*ATP2C1*		SEPT7	
	*EPHB1*		SERBP1	
	*ZIC4*		SESN1	
	*FXR1*		SF1	
	*C3orf70*		SF3B6	
	*PAQR3*		SFRP2	
	*PRDM8*		SFRP5	
	*EIF4E*		SFXN3	
	*KIAA1109*		SHISA4	
	*ADCY2*		SIL1	
	*MAP1B*		SIVA1	
	*MRPS27*		SKIL	
	*NREP*		SLC13A3	
	*EPB41L4A*		SLC20A2	
	*PRR16*		SLC23A4	
	*CTNNA1*		SLC24A4	
	*KCTD16*		SLC25A23	
	*CSNK1A1*		SLC27A1	
	*FAXDC2*		SLC2A10	
	*TENM2*		SLC35F6	
	*UIMC1*		SLC37A3	
	*RGS14*		SLC38A1	
	*TMED9*		SLC3A2	
	*SQSTM1*		SLC6A11	
	*MAPK9*		SLC6A9	
	*RREB1*		SLC7A10	
	*PHACTR1*		SLC7A6	
	*HIST1H2BD*		SLCO4A1	
	*HLA-B*		SLITRK2	
	*FANCE*		SMARCD2	
	*MED20*		SMC1A	
	*BYSL*		SNCAIP	
	*BAI3*		SNX3	
	*FAM135A*		SORBS1	
	*RIMS1*		SOX4	
	*EPHA7*		SRC	
	*HACE1*		SREBF1	
	*C1GALT1*		SREBF2	
	*POU6F2*		SRGAP2	
	*BCL7B*		SSBP4	
	*C7orf62*		ST6GALNAC4	
	*LRWD1*		STARD9	
	*RASA4*		STAU1	
	*POLR2J2*		STRADA	
	*BCAP29*		STRBP	
	*IMMP2L*		STT3B	
	*CPED1*		STXBP5	
	*LRRC4*		SUGP1	
	*PTN*		TACO1	
	*CCAR2*		TBCK	
	*CLU*		TCF25	
	*ANK1*		TCF7L2	
	*TGS1*		TDRP	
	*TERF1*		TEF	
	*UBE2W*		TELO2	
	*OTUD6B*		TERF2IP	
	*UQCRB*		TFB2M	
	*RIMS2*		TFCP2	
	*ELAVL2*		TGFBRAP1	
	*POLR1E*		TGIF2	
	*TOMM5*		TLE6	
	*STX17*		TMCC1	
	*TNC*		TMEM100	
	*NEK6*		TMEM128	
	*GLE1*		TMEM240	
	*NTNG2*		TMEM248	
	*FCN1*		TMEM251	
	*QSOX2*		TMEM44	
	*NELFB*		TMEM74B	
	*PDK3*		TMEM82	
	*ARHGEF6*		TMEM88B	
	*VAMP3*		TMEM94	
	*PER3*		TMPO	
	*DDI2*		TNFAIP8L1	
	*COL16A1*		TNK2	
	*BAI2*		TNKS2	
	*PDZK1IP1*		TOB2	
	*CMPK1*		TOM1L2	
	*MAN1A2*		TOMM6OS	
	*TARS2*		TOX	
	*ECM1*		TPR	
	*ARNT*		TPRKB	
	*RFX5*		TRAF5	
	*RIIAD1*		TRIL	
	*MRPL9*		TRIM2	
	*TDRKH*		TRIM32	
	*HRNR*		TRIM9	
	*ILDR2*		TRMT61A	
	*GPA33*		TRP53INP1	
	*NAV1*		TSC22D3	
	*LMOD1*		TSN	
	*CSGALNACT2*		TSPAN4	
	*ADO*		TSPAN9	
	*POLL*		TSPYL1	
	*TMEM180*		TUBGCP2	
	*ARL3*		TXNDC15	
	*CNNM2*		UBAP1	
	*CALHM2*		UBXN7	
	*CALHM1*		UCHL3	
	*LRRC27*		USP2	
	*KNDC1*		USP53	
	*PRKCDBP*		USP9X	
	*WEE1*		VPS37B	
	*NR1H3*		VWC2	
	*FAM180B*		WIPI1	
	*SPDYC*		WNK3	
	*LOC728975*		WNT7B	
	*CTSW*		WSB1	
	*C11orf73*		WWC1	
	*TYR*		XRCC5	
	*ALKBH8*		ZDHHC16	
	*ELMOD1*		ZEB1	
	*SPATA19*		ZFHX4	
	*JAM3*		ZFP26	
	*LMBR1L*		ZFP27	
	*POC1B*		ZFP318	
	*ACACB*		ZFP362	
	*NAA25*		ZFP46	
	*HECTD4*		ZFP503	
	*CCDC62*		ZFP518B	
	*ABCB9*		ZFP553	
	*ARL6IP4*		ZFP566	
	*ARHGEF40*		ZFP598	
	*OR5AU1*		ZFP747	
	*DAAM1*		ZFP764	
	*CCDC175*		ZFP846	
	*RTN1*		ZHX1	
	*TRMT61A*		ZKSCAN3	
	*ZNF280D*		ZMIZ1	
	*SKOR1*		ZNFX1	
	*TMEM8A*		ZRANB2	
	*PIGQ*		ZSCAN20	
	*FAM195A*		ZW10	
	*RHBDL1*		ZZZ3	
	*PRSS33*			
	*ZG16B*			
	*PDXDC1*			
	*NTAN1*			
	*RRN3*			
	*QPRT*			
	*TBX6*			
	*NDRG4*			
	*SLC38A7*			
	*ADAMTS18*			
	*GFAP*			
	*HOXB2*			
	*HOXB6*			
	*LRRC45*			
	*CCDC57*			
	*HOOK2*			
	*RTBDN*			
	*DNASE2*			
	*GCDH*			
	*MEF2BNB*			
	*TM6SF2*			
	*MAU2*			
	*LSR*			
	*MAG*			
	*CD22*			
	*FFAR1*			
	*COX7A1*			
	*ZFP82*			
	*ZNF566*			
	*ZNF260*			
	*ZNF529*			
	*ZNF567*			
	*ZNF850*			
	*ZNF781*			
	*ZNF607*			
	*NCCRP1*			
	*APOC1*			
	*ZNF544*			
	*ZNF584*			
